# EvolQG - An R package for evolutionary quantitative genetics

**DOI:** 10.12688/f1000research.7082.3

**Published:** 2016-11-08

**Authors:** Diogo Melo, Guilherme Garcia, Alex Hubbe, Ana Paula Assis, Gabriel Marroig

**Affiliations:** 1 Departamento de Genética e Biologia Evolutiva, Instituto de Biociências, Universidade de São Paulo, São Paulo, Brazil; 2 Departamento de Oceanografia, Instituto de Geociências, Universidade Federal da Bahia, Salvador, Brazil

**Keywords:** P-matrix, G-matrix, multivariate evolution, drift, morphological evolution, directional selection, matrix comparison, covariance matrix

## Abstract

We present an open source package for performing evolutionary quantitative genetics analyses in the R environment for statistical computing. Evolutionary theory shows that evolution depends critically on the available variation in a given population. When dealing with many quantitative traits this variation is expressed in the form of a covariance matrix, particularly the additive genetic covariance matrix or sometimes the phenotypic matrix, when the genetic matrix is unavailable and there is evidence the phenotypic matrix is sufficiently similar to the genetic matrix. Given this mathematical representation of available variation, the \textbf{EvolQG} package provides functions for calculation of relevant evolutionary statistics; estimation of sampling error; corrections for this error; matrix comparison via correlations, distances and matrix decomposition; analysis of modularity patterns; and functions for testing evolutionary hypotheses on taxa diversification.

## Introduction

Quantitative genetics deals with the evolution and inheritance of continuous traits, like body size, bone lengths, gene expressions or any other inheritable characteristic that can be measured on a continuous scale, or which can be transformed to a continuous scale. This framework has been used in selective breeding and in describing the different sources of variation in natural populations, as well as understanding the interaction of evolutionary processes with this variation ^32^. Quantitative genetics has been successful in describing short term evolution, and is also useful in understanding diversification at a macroevolutionary level. The core development of modern evolutionary quantitative genetics started with the generalization of the univariate breeders equation to the multivariate response to selection equation, derived by Lande and also referred to as the Lande equation ^28, 55^. The Lande equation relates the evolutionary change in trait means of a given population (Δ *z*) to the interaction between the additive genetic variation ( **G**-matrix) of this population and the directional selection ( *β*) acting on this population. The additive genetic variation of a population is represented by a symmetric square matrix called the **G**-matrix, which contains the additive genetic variance of each trait on the diagonal and the additive genetic covariance between traits on the off-diagonal elements. From the Lande equation, Δ *z* = **G** *β*, we can see that different populations may present markedly different responses (Δ *z*) to the same directional selection ( *β*) simply because these populations have distinct **G**-matrices. Other evolutionary forces affecting populations are also influenced by available variation, e. g., based on the **G**-matrix it is possible to test if morphological differentiation of extant taxa is compatible with genetic drift or stabilizing selection (e.g., 2, 35). Thus, describing and understanding changes in standing variation among populations ^7, 31, 34^ as well as understanding constraints imposed by populations standing variation (e.g., 19, 33, 51, 58) are major elements in evolutionary quantitative genetics.

In this article we describe the **EvolQG** package, developed to deal with the evolutionary quantitative genetics questions addressed above in the R environment for statistical computing ^53^. Our goal was to provide a suite of tools in a single consistent source, and to standardize and facilitate the adoption of these tools.

## Measurement error estimation

Before estimating a **G**-matrix, it is important to evaluate the influence of measurement error in data collection, since measurement error can drastically bias further analyses ^11^. Measurement error can be estimated by measuring each individual at least twice and estimating the amount of variation associated with each individual, which is the measurement error, in relation to total variation (i.e., the sum of within and between individuals variation) using an analysis of variance. The proportion of variance associated with among individual variation, and not within individual variation, is called the repeatability ^30^. A repeatability of 1 means that no variation is associated with measurement error. The function **CalcRepeatability()** performs the calculation described in 30 for a set of multivariate traits measured at least twice for each individual.

## Matrix estimation

In the rest of this article we assume that the covariance matrix of interest has already been estimated by some procedure. This matrix can be a simple covariance of all the observed traits, or an estimated parameter from a more complicated linear model. The simplest case of a linear model approach would be using a multivariate analysis of covariance (MANCOVA) to control for differences in trait means that are not of immediate interest in the analyses (e.g., sexual dimorphism, geographic variation, etc.). The residual pooled within-group covariance matrix can be used in subsequent analysis ^34^. The **EvolQG** function **CalculateMatrix()** uses R’s **lm()** model object to calculate variance-covariance matrices adjusting for the proper degrees of freedom in a simple fixed-effects MANCOVA. More complicated methods may be used to obtain **G**-matrices, such as an animal model or a mixed model ^32, 57^, and these can be used for further analysis using **EvolQG**.

**EvolQG** also provides a simple Bayesian model for phenotypic matrix estimation using a conjugate inverse Wishart prior, implemented in the function **BayesianCalculateMatrix()**. The default behavior is to use a prior covariance matrix with the observed variances (from the data) on the diagonal and zeros in the off-diagonal elements. This method uses a type of regularization prior which provides a “shrinkage” posterior estimate which has some attractive properties when compared to the maximum likelihood estimate, such a lower mean squared error and a well behaved inverse ^29, 44, 59^. The maximum *a posteriori* estimate is calculated analytically, and optionally the function also provides a sample from the posterior distribution and the median of these samples. These samples can be used in other Bayesian methods described in the next sections, or to calculate confidence intervals for any analysis using the covariance matrices.

Ideally all the analysis we describe should be performed on **G**-matrices, but accurate **G**-matrix estimation can be hard, requiring large sample sizes, many families and known genealogies ^60^. One alternative we advocate for, which is usually more feasible, is to use the phenotypic covariance matrix (the **P**-matrix) as a proxy of the population’s **G**-matrix ^8, 56^. Conditions on where this approximation is reasonable depend on the structure of developmental and environmental effects, and testing for similarity is an empirical question that should be undertaken before using the **P**-matrix as a proxy for the **G**-matrix, ideally by direct comparison (e.g., 13). As a general rule, high similarity between populations’ **P**-matrices is a good indicator of high similarity between **P** and **G**, and of a stable shared **G**-matrix pattern, since the similarity between populations must come from either a common genetic structure, or the unlikely scenario of a different genetic structure buffered by an exactly compensating environmental structure in each population that leads to high similarity between phenotypic covariation.

Some of the methods described below are not applicable to covariance matrices, only to correlation matrices. Correlations are standardized measures of association that are bounded between [−1, 1], and, unlike covariances, can be directly compared for pairs of traits with different scales. In most instances, correlation matrices can be obtained directly from covariance matrices by using the R function **cov2cor()**.

## Matrix error and repeatabilities

A **G**-matrix will always be estimated with error ^20, 37, 41^, and it is important to take this error into account in further analyses. In some circumstances we want to compare two or more **G**-matrices, calculating the matrices correlations (see section **Matrix Comparison**). However, due to error in estimating these matrices, their correlations will never be one, even if the actual population parameter values are identical ^8^. Thus, matrix repeatabilities are used to correct matrix correlations by taking sampling error into account. The basic premise of all the methods is that taking repeated samples from the same population and comparing the resulting matrices would still give correlations that are lower than 1. We estimate the maximum correlation between matrices taken from the same population and correct the observed correlation by this maximum value. The corrected correlation between two observed matrices will be given by the original correlation divided by the geometric mean of their repeatabilities. If the repeatability of both matrices is one, the observed correlation does not change under the correction, and lower repeatabilities yield larger corrections. Estimating error for **G**- and **P**-matrices require different methods. The objective when estimating error is usually to create a sample of matrices that reflects our uncertainty regarding the estimated matrix. Because **G**-matrix are estimated using mixed models or animal models ^32, 57^ this model structure must be taken into account, and sampling strategies that work in P-matrices will severely underestimate error in **G**-matrices. Given these restrictions, we can generate a distribution of **G**-matrices in several ways: (i) mixed model packages often provide the functionality of bootstrapping over the exchangeable units in the design (i.e. permuting sires in a half-sib or full-sib design) and generate error estimates using these samples; (ii) Houle & Meyer ^23^ use an asymptotic approximation for the the parametric sampling distribution of the estimated **G**-matrix to generate samples and estimate error, a method called MVN-REML available in the program WOMBAT ^40^; (iii) in a fully Bayesian context, samples from the posterior distribution of the **G**-matrix can be used. These samples can then be used to calculate repeatabilities or confidence intervals for derived quantities. Using any of these sampling methods the repeatability can be estimated as the average comparison value for all pairs of sampled matrices or as the average comparison of the estimated matrix and the sampled matrices. 

When working with **P**-matrices things are simpler, and repeatabilities are straight forward to calculate using **EvolQG**. We provide a number of methods for estimating repeatability in **P**-matrices, and their results can be passed on to the functions that calculate matrix correlations (section **Matrix Comparison**):

**AlphaRep():** Cheverud ^8^ describes an analytical expression for the repeatability of a correlation matrix. This expression is asymptotically convergent, so it should be used only when sample sizes are large, at least larger than the number of traits.

**BootstrapRep():** We may estimate the repeatability of the covariance (or correlation) structure of a given data set using a bootstrap procedure, sampling individuals with replacement from the data set and calculating a covariance (or correlation) matrix from each sample. The mean value of the correlation between the random sample matrix and the original estimated matrix is an estimate of the repeatability. This method has the advantage of not assuming any distribution on the data, but does provide inflated estimates of repeatabilities for small data sets. Even so, upwardly biased matrix repeatabilities are not so problematic, because they lead to conservative corrections of matrix correlations. However, users should be aware of this bias and should not interpret a high repeatability obtained from a small data set as indicating that the parameter is well estimated.

**MonteCarloRep():** We can use the observed covariance matrix as the Σ parameter in a multivariate normal distribution, and produce samples from this distribution, using a fixed sample size. The covariance (or correlation) matrix for each sample is compared to the observed matrix, and the mean of these comparisons is an estimate of the repeatability ^34^. This method has the advantage of being easy to apply to matrices coming from linear models with many fixed effects, and not requiring the original data; but can also lead to inflated repeatabilities for small samples.

Both **MonteCarloRep()** and **BootstrapRep()** are based on generic functions ( **MonteCarloStat()** and **BootstrapStat()**) which can be used for general sampling from covariance matrices using normal distributions or to generate bootstrap samples from a set of individuals, respectively. These functions can be used to generate confidence intervals for **P**-matrices for any of the evolutionary statistics described below, or any user defined function.

Sometimes the question we are trying to answer does not involve matrix comparisons, so other methods of assessing and correcting for error are needed.

**Rarefaction():** Rarefaction consists of taking progressively smaller samples with replacement from the original data set, calculating some statistic on each data set and comparing this with the full data set. These comparisons gives a general idea of how the inferences would change if we had smaller sample sizes, and how robust our data set is with respect to sampling, given that an appropriately large initial sample is available. The default operation is to calculate the covariance or correlation matrices and compare them using any of the matrix comparison methods (see section **Matrix Comparison**). The generic **Rarefaction-Stat()** can also be used to generate rarefaction curves for any user defined function.

**ExtendMatrix():** Marroig *et al.* ^37^ showed that sampling error on covariance matrix estimation can have a dramatic effect on the reconstruction of net selection gradients using the multivariate response to selection equation ^28^. One way to improve estimates is the simple procedure of "extending" the eigenvalues of the covariance matrix, where all the eigenvalues lower than a certain threshold are substituted by the smallest eigenvalue above the threshold. This substitution causes minimal changes in the distribution of phenotypes, but improves dramatically the estimates of net selection gradients. Alternatives to the extension method involve using regularized estimators for the covariance matrix, like Bayesian or shrinkage estimators ^29, 59^, available from package **corpcor**. See 37 for a thorough examination of the performance and consequences of the extension and shrinkage methods on simulated and real data sets.

## Evolutionary statistics

Hansen & Houle ^19^ provide a suite of statistics that have fairly good biological interpretations for a given **G**- or **P**-matrix. Marroig *et al.* ^38^ is a comprehensive example of how these statistics may be used for interpreting morphological data.

The function **MeanMatrixStatistics()** calculates most of these statistics and their distributions, as shown below. **MonteCarloStat()** and **BootstrapStat()** can be used to generate confidence intervals for these statistics when using **P**-matrices. Also, the previously available R package **evolvability** ^5^ implements some of these functions and provides confidence intervals.

**Figure 1.**
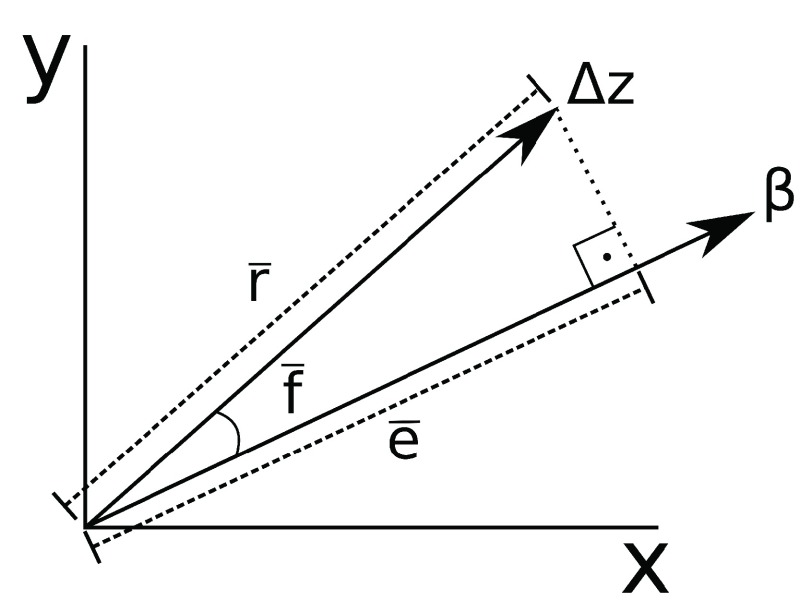
Graphical representation of evolvability $\left(\bar{e}\right),$ respondability $\left(\bar{r}\right)$ and flexibility $\left(\bar{f}\right)$ for a single selection gradient ( *β*) and the corresponding response (Δ *z*) in the two dimensions defined by traits *x* and *y*.

In the following, *E*[·] _*β*_ represents the expected value over many random *β* vectors with unit norm, < ·, · > represents the dot product between two vectors, *cos*(·, ·) is the cosine between two vectors, **G** is an arbitrary covariance matrix, **G** ^−1^ is the inverse **G**, *tr*( **G**) is the trace of **G**, and ॥ · ॥ the Euclidean norm of a vector. **MeanMatrixStatistics()** calculates:

• Mean squared correlation ( *r* ^2^): Given a correlation matrix, the elements below the diagonal are squared and averaged resulting in a measure of integration, that is, overall association between traits (also see the section **Modularity and Integration** and 49).
• Coefficient of variation of eigenvalues (ICV): A measure of integration that is suitable for covariance matrices, as it takes the amount of variation into account. Notice that at least for mammals, mean squared correlations and ICV generally have high correlation, but can lead to different conclusions if the traits included in the analysis have very different variances (due to scale, for example). If σ _*λ*_ is the standard deviation of the eigenvalues of a covariance matrix, and $\overset{–}{\lambda }$ is the mean of the eigenvalues, the ICV is:
$ICV=\frac{\sigma_{\lambda }}{\overset{–}{\lambda }}$
• Percent of variation in first principal component: If $\lambda_{1}^{G}$ is the leading eigenvalue of **G**, we calculate this percentage as:
$PC1\%=\frac{\lambda_{1}^{G}}{tr(G)}$
• Evolvability ( Figure 1): The mean projection of the response to random selection gradients with unit norm onto the selection gradient. This projection is a measure of a population’s available variation in the direction of a particular selection gradient, averaged across all directions ^19^.
  $$\bar{e}=E{\left[<G\beta ,\beta >\right]}_{\beta }$$
• Flexibility ( Figure 1): The mean cosine of the angle between random selection gradients and the corresponding responses. Flexibility measures on average how the response to selection aligns with the selection gradient ^38^.
  $$\bar{f}=E{\left[\cos\left(G\beta ,\beta \right)\right]}_{\beta }$$
• Respondability ( Figure 1): Mean norm of the response to random selection gradients with unit norm. It also estimates how fast the population mean will change under directional selection ^19, 38^.
  $$\bar{r}=E{\left[\|G\beta \|\right]}_{\beta }$$
• Conditional Evolvability: Measures the mean response to selection in the direction of a given *β* when other directions are under stabilizing selection ^19^.
  $$\bar{c}=E{\left[{\left(<G^{-1}\beta ,\beta >\right)}^{-1}\right]}_{\beta }$$
• Autonomy: Measures the proportion of variance in the direction of a given *β* that is independent from variation in other directions. Therefore, mean Autonomy can also be calculated as the mean ratio between Conditional Evolvability $\left(\bar{c}\right)$ and Evolvability $\left(\bar{e}\right)$ ^19^.
  $$\bar{a}=E{\left[{\left(<G^{-1}\beta ,\beta >\right)}^{-1}{\left(<G\beta ,\beta >\right)}^{-1}\right]}_{\beta }$$
• Constraints: The mean correlation between the response vector to random selection gradients and the matrix’s first principal component ^38^. If $\Lambda_{1}^{G}$ is the first principal component of **G**, constraints are measured as:
  $$E{\left[\cos\left(G\beta ,\Lambda_{1}^{G}\right)\right]}_{\beta }$$

## Matrix comparison

A **G**-matrix describes how the variation in particular populations is structured, but frequently the relevant question is how similar or dissimilar two populations are with respect to this standing variation. Because no two populations are identical, different patterns of variation are the norm. Depending on the evolutionary question at hand, different methods of comparing variation may be required. One possible application of matrix comparisons is when we wish to apply the Lande equation to micro and macroevolution, because doing so requires some additional assumptions, such as a relative stability of the **G**-matrix over generations ^24^. Comparing extant covariance matrices is a test of this required stability (e.g. 34). For a thoughtful discussion on the biological relevance of statistical significance in matrix comparisons, see the discussion in 16.

### Matrix correlations

One approach to estimate the similarity or dissimilarity between matrices is to calculate the correlation between these matrices. **EvolQG** provides several functions for pairwise matrix correlation.

**RandomSkewers():** The Random Skewers (RS) method makes use of the Lande equation ^28^, Δ *z* = *Gβ*, where Δ *z* represents the vector of response to selection, **G** the **G**-matrix and *β* the directional selection vector, or selection gradient. In the RS method, the two matrices being compared are multiplied by a large number of normalized random selection vectors, and the resulting response vectors to the same selection vector are compared via a vector correlation (the cosine between the two vectors). The mean value of the correlation between the responses to the same selective pressure is used as a simple statistic of how often two populations respond similarly (in the same direction) to the same selective pressure:

$$RS\left(A,B\right)=E{\left[\cos\left(A\beta ,B\beta \right)\right]}_{\beta }\;(1)$$

Where *E*[·] _*β*_ is the expected value over random selection vectors *β*. Significance in the random skewers comparison can be determined using a null expectation of correlation between random vectors. If the observed correlation between two matrices is above the 95% percentile of the distribution of correlations between random vectors, we consider the correlation significant and infer that there is evidence the two populations behave similarly under directional selection. Other implementations of the RS method sometimes resort to other forms of calculating significance, such as generating random matrices and creating a random distribution of correlations between matrices. Generating this distribution is difficult to do because generating random matrices with the properties of biological covariance structures is hard, see the **RandomMatrix()** function for a quick discussion on this problem. But perhaps more important than the significance test, the RS method measure the overall similarity of the responses predicted by the two matrices under directional selection, a property that is at the core of applications of quantitative genetics theory to evolutionary problems. In this sense, a significant similarity measure by Random Skewers might still be low for a given application, when small differences in the direction of response are important. The RS values range between -1 (the matrices have opposite structures) and 1 (the matrices share the same structure), and zero means the matrices have distinct structures.

**MantelCor():** Correlation between matrices can be done using a simple Pearson correlation between the corresponding elements. Significance of this comparison must take the structure into account, so it is calculated by a permutation scheme, in which a null distribution is generated by permutation of rows and columns in one of the matrices and repeating the element-by-element correlation (i.e. Mantel test). The observed correlation is significant when it is larger than the 95% quantile of the permuted distribution. This method should only be used in correlation matrices, and cannot be used on covariance matrices because the variances might be very different, leading to large differences in the scale of the covariances. This scale difference can lead to a massive inflation in the correlation between matrices. The correlation between matrices range between -1 and 1, and higher correlations indicate matrices have more similar structures, null correlations indicate the matrices have distinct correlation structures. Correlations near zero can also occur if the elements of the matrices have nonlinear relations between them, as in all Pearson correlations. Negative correlations indicate the pattern of association between traits is reversed in the two matrices.

**KzrCor():** The Krzanowski shared space, or Krzanowski correlation, measures the degree to which the first principal components (eigenvectors) span the same subspace ^3, 26^, and is suitable for covariance or correlation matrices. If two *n × n* matrices are being compared, the first $k=\frac{n}{2}-1$ principal components from one matrix are compared to the first *k* principal components of the other matrix using the square of the vector correlations, and the sum of the correlations is a measure of how congruent the spanned subspaces are. We can write the Krzanowski correlation in terms of the matrices’ principal components ( $\Lambda_{i}^{A}$ being the i *th* principal component of matrix *A*):

$$KrzCor(A,B)\;=\;\frac{1}{k}\sum_{i=1}^{k}\sum_{j=1}^{k}cos^{2}(\Lambda_{i}^{A},\Lambda_{j}^{B})\;(2)$$

The Krzanowski correlation values range between 0 (two subspaces are dissimilar) and 1 (two subspaces are identical).

**PCAsimilarity():** The Krzanowski correlation compares only the subspace shared by roughly the first half of the principal components, but does not consider the amount of variation each population has in these directions of the morphological space ^62^. In order to take the variation into account, we can add the eigenvalue associated with each principal component into the calculation, effectively weighting each correlation by the variance in the associated directions. If $\lambda_{i}^{A}$ is the i *th* eigenvalue of matrix A, we have:

$$\begin{array}{rr} PCAsimilarity(A,B)=\frac{\sum_{i=1}^{n}\sum_{j=1}^{n}\lambda_{i}^{A}\lambda_{j}^{B}\cos^{2}(\Lambda_{i}^{A},\Lambda_{j}^{B})}{\sum_{i=1}^{n}\lambda_{i}^{A}\lambda_{j}^{B}} & \;(3) \end{array}$$

Note the sum spans all the principal components, not just the first *k* as in the Krzanowski correlation method. This method gives correlations that are very similar to the RS method, but is much faster. The PCA similarity values range between 0 (the shared subspaces have no common variation) and 1 (the shared subspaces have identical variation).

### Matrix decomposition

Some methods attempt to characterize how a set of matrices differ, going beyond simple correlations. **EvolQG** provides efficient implementations of some of these methods.

**SRD():** The RS method can be extended to give information into which traits contribute to differences in terms of the pattern of correlated selection due to covariation between traits in two populations ^36^. The Selection Response Decomposition does this by treating the terms of correlated response in the Lande equation as separate entities. Writing out the terms in the multivariate response to selection equation:

$$\begin{array}{l} A\beta =\left(\begin{array}{llll} A_{11} & A_{12} & \cdots & A_{1n}\\ A_{21} & A_{22} & \cdots & A_{2n}\\ \vdots & \vdots & \ddots & \vdots \\ A_{n1} & A_{n2} & \cdots & A_{nn} \end{array}\right)\left(\begin{array}{l} \beta_{1}\\ \beta_{2}\\ \vdots \\ \beta_{n} \end{array}\right)=\;(4)\\ =\left(\begin{array}{l} A_{11}\beta_{1}+A_{12}\beta_{2}+\cdots +A_{1n}\beta_{n}\\ A_{21}\beta_{1}+A_{22}\beta_{2}+\cdots +A_{2n}\beta_{n}\\ \vdots \\ A_{n1}\beta_{1}+A_{n2}\beta_{2}+\cdots +A_{nn}\beta_{n} \end{array}\right)=\Delta z \end{array}$$

Separating the terms in the sums of the right hand side:

$$\begin{array}{llll} (A_{11}\beta_{1} & A_{12}\beta_{2} & \cdots & A_{1n}\beta_{n})\\ (A_{21}\beta_{1} & A_{22}\beta_{2} & \cdots & A_{2n}\beta_{n})\\ \vdots & \vdots & \ddots & \vdots \\ (A_{n1}\beta_{1} & A_{n2}\beta_{2} & \cdots & A_{nn}\beta_{2}) \end{array}\;(5)$$

Each of these row vectors $r_{i}^{A\beta }={\left(A_{ij}\beta_{j}\right)}_{j=1\ldots n}$ are the components of the response to the selection gradient *β* on trait *i*. The term *A _ii_β _i_* represents the response to direct selection on trait *i*, and the terms ( *A _ij_β _j_*) _*i*≠ *j*_ represent the response to indirect selection due to correlation with the other traits. Given two matrices, *A* and *B*, we can measure how similar they are in their pattern of correlated selection on each trait by calculating the correlation between the vectors *r _i_* for each trait for random selection vectors of unit norm. The mean SRD score for trait *i* is then:

$$\mu SRD{\left(A,B\right)}_{i}=E{\left[cor\left(r_{i}^{A\beta },r_{i}^{B\beta }\right)\right]}_{\beta }\;(6)$$

And the standard deviation of the correlations gives the variation in SRD scores:

$$\sigma SRD{\left(A,B\right)}_{i}=\sqrt{E{\left[{\left(cor\left(r_{i}^{A\beta },r_{i}^{B\beta }\right)-\mu SRD_{i}\right)}^{2}\right]}_{\beta }}\;(7)$$

When the same trait in different matrices share a correlated response pattern, *µSRD* is high and *σSRD* is low; if the correlated response pattern is different, *µSRD* is low and *σSRD* is high. See 36 for details and examples.

**RSprojection():** Aguirre *et al.* ^3^ used a modification of the Random Skewers method to examine differences in the magnitude of variation in different directions in a set of covariance matrices. This method is most useful when a posterior sample of covariance matrices is available, as this sample allows us to identify directions in the morphospace that show relevant differences in the amount of variation between any two matrices. The function **RSprojection()** uses a set of posterior samples to identify the directions that represent the most significant differences in variance, and to compare the amount of variation in each matrix for each of these directions. The posterior samples of covariance matrices can be obtained using any Bayesian linear model package, such as MCMCglmm ^17^, or, for simple cases, **BayesianCalculateMatrix()**. This method identifies directions where there is a significant difference in the amount of variation, so it is most useful when the matrices being compared have similar scales, since matrices with identical structure might have different amounts of variation in all directions simply because the total amount of variation is larger in one of the matrices.

**Figure 2.**
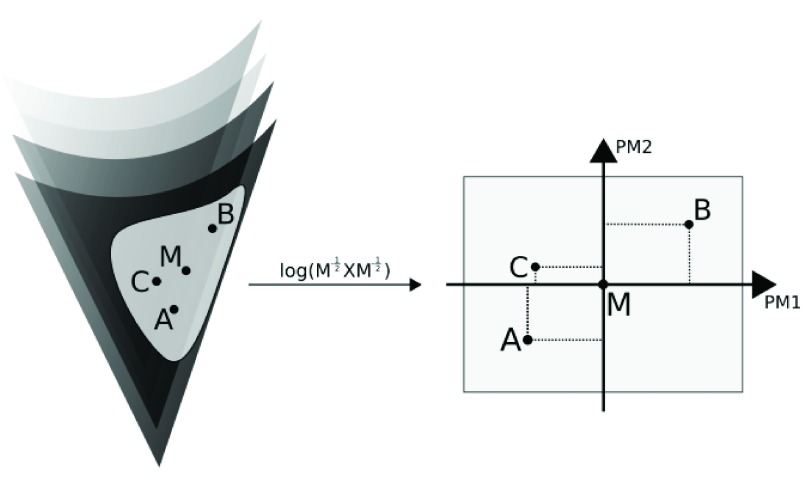
Graphical depiction of the eigentensor decomposition of covariance matrices A, B and C. The mean matrix M is estimated within the non-Euclidean space of symmetric positive-definite matrices; the transformation $f(X)=\mathrm{Log}(M^{-\frac{1}{2}}{\text{XM}}^{-\frac{1}{2}})$ maps A, B and C into an Euclidean space centered on M. Only in this Euclidean space are the eigentensors (PM1 and PM2) estimated.

**EigenTensorDecomposition():** Hine *et al.* ^21^ proposes using covariance tensors for characterizing covariance matrix variation, based on Basser & Pajevic ^4^. Covariance tensors can be further decomposed into orthogonal eigentensors and eigenvalues; each covariance matrix in the sample can thus be represented as a combination of such eigentensors, in a manner analogous to Principal Component Analysis. Considering the non-Euclidean (Riemannian) nature of the space of symmetric positive-definite matrices ( Figure 2), the implementation of this procedure in **EvolQG** relies on estimating a geometric mean matrix (which minimizes the sum of Riemannian distances among observations ^43^; implemented as **MeanMatrix()**) and mapping such observations into an actual Euclidean space using the function

$$f(X)=Log(M^{-\frac{1}{2}}XM^{-\frac{1}{2}})\;(8)$$

where **M** refers to the estimated geometric mean matrix and *Log* refers to the matrix logarithm operator. Only then a covariance tensor can be estimated, considering that the estimation of orthogonal eigentensors assumes that observations are contained within an Euclidean space, equipped with definitions for both angle and distance. The function **EigenTensorDecomposition()** implements these steps; furthermore, given an eigentensor decomposition estimated using this function, it is possible to project other covariance matrices of the same size as the original sample onto the obtained eigentensors ( **ProjectMatrix()**), and also reconstruct covariance matrices based on their scores over eigentensors ( **RevertMatrix()**), which may be useful for observing the direction associated with each eigentensor as actual covariance matrices. This function uses the inverse operation to equation 8, that is

$$f^{-1}(X)=M^{\frac{1}{2}}Exp(X)M^{\frac{1}{2}})\;(9)$$

where *Exp* refers to the matrix exponential operator. This operation maps the matrix **X** back onto the non-Euclidean space of symmetric positive-definite matrices. If a posterior sample of covariances matrices is available, this function can be used to implement the hypothesis-testing method described in Aguirre *et al.* ^3^ using covariance tensors, but this test also requires a null distribution of matrices that has to be tailored to the data at hand. Also, since the covariance tensor is calculated using the covariation between elements in the covariance matrix, these methods require two levels of replication in the data: individuals within populations for the calculation of the covariance matrices, and several populations for the calculation of the covariance tensor. This requirement should be kept in mind when interpreting the results, as small samples at either level can lead to poorly estimated parameters and misleading results.

### Matrix distances

Another approach to estimate the similarity or dissimilarity between matrices is to calculate the distance between a pair of matrices. Matrices distances are different from correlations in that correlations are limited to [−1, +1], while distances must only be positive. Also, smaller values of distances mean more similarity. Two distances are in use in the current evolutionary literature, and are implemented in the function **MatrixDistance()**.

- Overlap distance: Ovaskainen *et al.* ^48^ proposed a distance based on probability distributions, where two covariance matrices would have a distance proportional to how distinguishable they are. This distance is natural if we think of covariance matrices as describing the probability distribution of phenotypes or additive values in the population. The higher the probability of a random draw coming from the distribution defined by one of the matrices being misclassified as coming from the distribution defined by the other, the lower the distance. For two probability distributions *f* and *g*, the probability of misclassifying a draw from *f* as coming from *g* is:
  $$q\left(f,g\right)=\int_{R^{n}}\frac{g(x)}{f(x)+g(x)}f(x)dx\;(10)$$
  where *n* is the dimensionality of the space in which the distributions are defined. If the distributions are indistinguishable, *q*( *f*, *g*) = 1/2, if they are completely distinguishable *q*( *f*, *g*) = 0. We can then define the distance as:
  $$d(f,g)=\sqrt{1-2q(f,g)}\;(11)$$
  Since *q*( *f*, *g*) is symmetrical, *d*( *f*, *g*) is also symmetrical, and the square root guaranties that *d*( *f*, *g*) satisfies the triangle inequality ^48^. Calculation is straight forward and can be done with a simple sampling Monte Carlo scheme, see 48 for details.
- Riemann distance: Mitteroecker and Bookstein ^42^ use a Riemannian metric in the space of positive definite matrices (either covariance or correlation matrices), based on exponential mapping ^43^ to quantify transition in the ontogenetic trajectory of phenotypic covariance matrices. This metric is based on the eigenvalues of the product of one matrix to the inverse of the other. If *λ _i_* are the eigenvalues of *A* ^−1^ *B* (or *AB* ^−1^), we have:
  $${\|A,B\|}_{\mathrm{cov}}=\|B,{A\|}_{\mathrm{cov}}=\sqrt{\sum_{i=1}^{p}{\left[\log\left(\lambda_{i}\right)\right]}^{2}}\;(12)$$
  This distance has the advantage of being invariable under changes in the base used to represent the matrices. See 42 for a discussion on the biological relevance of this distance.

## Phylogenetic comparisons

**PhyloW():** Given a set of covariance matrices for the terminal taxa in a phylogeny, we can estimate the covariance matrix for internal nodes by taking means over sister taxa, weighted by sample size. The mean matrix at the root node is the within-group covariance matrix in a MANCOVA with the terminal clades as the fixed effects. **PhyloW()** does this estimation by taking a tree and a set of measurements (covariance matrices) and returns means weighted by sample size for internal nodes. The implementation is generic, so this function can also be used to calculate weighted means for any numerical measurement with an addition operation implemented in R.

For G-matrices it might not be clear what the appropriate weight to use in each node, as the linear mixed models used in G-matrix estimation do not assign a clear degree of freedom for the covariance matrix. Number of families could plausibly be a good weighing factor, but one should proceed with caution, or use a more direct approach of including the phylogeny in G-matrix estimation.

While using the within-group covariance matrix is a reasonable alternative as the estimator of an ancestral covariance matrix, it ignores branch lengths, and so should be used carefully when matrix differences are correlated to phylogenetic distance. An alternative when matrix evolution depends of branch lengths is to reconstruct every position of the covariance matrix independently via maximum likelihood, but this method can result in non positive-definite estimates.

**PhyloCompare():** Sometimes it is not practical to pairwise compare every single population in a study, since for a large number of populations these results can be difficult to interpret. In these cases, comparing populations in a phylogeneticaly structured way can be helpful in detecting major transitions or differences between clades. **PhyloCompare()** takes estimates for all the nodes in a tree and compares sister groups by any comparison method, providing comparison values for every inner node.

## Hypothesis testing

### Modularity and integration

Modularity is a general concept in biology, and refers to a pattern of organization that is widespread in many biological systems. In modular systems, we find that some components of a given structure are more related or interact more between themselves than with other components. These highly related groups are termed modules. The nature of this interaction will depend on the components being considered, but may be of any kind, like physical contact between proteins, joint participation of enzymes in given biochemical pathways, or high correlation between quantitative traits in a population. This last kind of modularity is called variational modularity, and is characterized by high correlations between traits belonging to the same module and low correlation between traits in different modules ^61^. In the context of morphological traits, variational modularity is associated with the concept of integration ^47^, that is, the tendency of morphological systems to exhibit correlations due to common developmental factors and functional demands ^9, 18^.

Both modularity and integration may have important evolutionary consequences, since sets of integrated traits will tend to respond to directional selection in an orchestrated fashion due to genetic correlations between them; if these sets are organized in a modular fashion, they will also respond to selection independently of one another ^38^. At the same time, selection can alter existing patterns of integration and modularity, leading to traits becoming more or less correlated ^24, 39^. The correlations between traits in a **G**-matrix then carries important information on the expected response to selection and on the history of evolutionary change of a given population.

**TestModularity():** Variational modularity can be assessed by comparing a modularity hypothesis (derived from development and functional considerations) with the observed correlation matrix. If two traits are in the same variational module, we expect the correlation between them to be higher than between traits belonging to different modules. We test this partition by creating a modularity hypothesis matrix and comparing it via Pearson correlation between the corresponding elements in the observed correlation matrix. The modularity hypothesis matrix consists of a binary matrix where each row and column corresponds to a trait. If the trait in row *i* is in the same module of the trait in column *j*, position ( *i*, *j*) in the modularity hypothesis matrix is set to one, if these traits are not in the same module, position ( *i*, *j*) is set to zero. Significant correlation between the hypothetical matrix representing a modularity hypothesis and the observed correlation matrix represents evidence of the existence of this variational module in the population. We also measure the ratio between correlations within a module (AVG+) and outside the module (AVG-). This ratio (AVG+/AVG-) is called the AVG Ratio, and measures the strength of the within-module association compared to the overall association for traits outside the module. The higher the AVG Ratio, the bigger the correlations within a module in relation to all other traits associations in the matrix (e.g., 50). **TestModularity()** also provides the Modularity Hypothesis Index, which is the difference between AVG+ and AVG- divided by the coefficient of variation of eigenvalues. Although the AVG Ratio is easier to interpret (how many times greater the within-module correlation is compared to the between-module correlation) than the Modularity Hypothesis Index, the AVG Ratio cannot be used when the observed correlation matrix presents correlations that differ in sign, and this is usually the case for residual matrices after size removal (for example with **RemoveSize()**, but see 25 for other alternatives). In these cases, the Modularity Hypothesis Index is useful and allows comparing results between raw and residual matrices ^51^.

**LModularity():** If no empirical or theoretical information is available for creating a modularity hypothesis, such as functional or developmental data, we can try to infer the modular partition of a given population by looking only at the correlation matrix and searching for the trait partition that minimizes some indicator of modularity. Borrowing from network theory, we can treat a correlation matrix as a fully connected weighted graph, and define a Newman-like modularity index ^45^. If *A* is a correlation matrix we define *L* modularity as:

$$L=\sum_{i\neq j}\left[A_{ij}-\frac{k_{i}k_{j}}{2m}\right]\delta (g_{i},g_{j})\;(13)$$

The terms *g _i_* and *g _j_* represent the partition of traits, that is, in what modules the traits *i* and *j* belong to. The function *δ*( *·*, *·*) is the Kronecker delta, where:

$$\delta (x,y)=\{\begin{array}{ll} 1 & \text{if}\;x=y\\ 0 & \text{if}\;x\neq y \end{array}\;(14)$$

This means only traits in the same module contribute to the value of *L*. The term *k _i_* represent the total amount of correlation attributed to trait *i*, or the sum of the correlation with trait *i*:

$$k_{i}=\sum_{j\neq i}A_{ij}\;(15)$$

And *m* is the sum of all $k\;(m=\sum_{i}k_{i}).$ The term $\frac{k_{i}k_{j}}{2m}$ plays the role of a null expectation for the correlation between the traits *i* and *j*. This choice for the null expectation is natural when we impose that it must depend on the values of *k _i_* and *k _j_* and must be symmetrical ^45^. So, traits in the same module with correlations higher than the null expectation will contribute to increase the value of *L*, while traits in the same module with correlation less than the null expectation will contribute to decrease *L*. With this definition of *L*, we use an optimization procedure to find the partition of traits (values of *g _i_*) that maximizes *L*. This partition corresponds to the modularity hypothesis inferred from the correlation matrix, and the value of *L* is a measure of modularity comparable to the AVG Ratio. The igraph package ^10^ provides a number of community detection algorithms that can be used on correlation matrices using this function.

**RemoveSize():** If the first principal component of a covariance or correlation matrix corresponds to a very large portion of its variation, and all (or most) of the entries of the first principal component are of the same sign (a *size* principal component, see 33), it is useful to look at the structure of modularity after removing this dominant integrating factor. This removal is done using the method described in 6. Porto *et al.* ^51^ show that modularity is frequently more easily detected in matrices where the first principal component variation was removed and provide biological interpretations for these results.

### Drift

Selection is frequently invoked to explain morphological diversification, but the null hypothesis of drift being sufficient to explain current observed patterns must always be entertained. We can test the plausibility of drift for explaining multivariate diversification by using the regression method ^1^, or the correlation of principal component scores ^1, 35^. Since both these tests use drift as a null hypothesis, failure to reject the null hypothesis is not evidence that selection was not involved in the observed pattern of diversification, only that the observed pattern is compatible with drift. Also, these methods assume that the matrices involved share some degree of similarity, and should ideally be proportional to each other. We would be very weary of using these methods if the matrices are too dissimilar, or if the results change radically if different matrices are used as the ancestral matrix. Also, these tests rely on two levels of replication, taxa and traits. As a general guideline, at least 20 traits and at least 8 taxa should be sampled for using these methods with any confidence, and results should be analyzed in conjunction with other lines of evidence.

**DriftTest()**: Under drift and without gene flow, we expect that the current between group variance for many populations will be proportional to the ancestral population’s covariance structure, which is approximated by the pooled within-group covariance matrix. This test assumes matrices remain proportional to the ancestral matrix, but this might not always be the case ^14^. Conditions for the validity of these assumptions are reviewed in 52, and matrices for the extant groups should always be tested for similarity. Under these conditions, if *B* is the between group covariance matrix, and *W* is the within group covariance matrix, *t* is the time in number of generations and *N _e_* is the effective population size, we have:

$$B\;\propto \;(t/N_{e})W\;(16)$$

If we express all these matrices in terms of the eigenvectors of *W*, so that *W* is diagonal, we can write *B* as the variance of the scores of the means on these eigenvectors. The relationship between *B* and *W* can be expressed as a log regression, where *B _i_* is the variance between groups in the projected means and $\lambda_{i}^{W}$ are the eigenvalues of *W*:

$$\log(B_{i})=\log(t/N_{e})+\beta \log(\lambda_{i}^{W})\;(17)$$

where *β* is the regression coefficient. Under drift we expect *β* to be one. If *β* is significantly different from one, we have evidence that drift is not sufficient to explain currently observed diversification.

**MultivDriftTest()**: This drift test verifies the plausibility of drift in a multivariate context when only two populations are available, one ancestral (or reference) and one derived. Let *z*_0_ represent a vector of means from *m* traits in an ancestral population. After *t* generations, the expected traits mean for *n* populations under drift would correspond to *z*_0_ with variance given by *B* = ( *t/N _e_*) *W*, where *B* represents the expected between group covariance matrix, *W* is the genetic covariance matrix from the ancestral (or reference) population, and *N _e_* is the effective population size ^22, 27, 28^. So, given the ancestral population mean and *G*-matrix, we can use this model to estimate the *B*-matrix expected under drift. We can then use this *B*-matrix as the Σ parameter in a multivariate normal distribution and sample *n* populations from this distribution. Using this sample of random populations, we can assess the amount of divergence expected by drift, estimated as the norm of the difference vectors between ancestral (or reference) and simulated population means. Then, we can compare the observed amount of divergence between the ancestral and derived populations, calculated as the norm of the difference vector between them, taking into account the standard error of traits means. An observed divergence higher than the expectations under drift indicates that genetic drift is not sufficient to explain currently observed divergence, suggesting a selective scenario.

**PCScoreCorrelation():** This test of drift relies on the correlation between principal component scores of different populations. Under drift alone, we expect the mean scores of different populations in the principal components of the within-group covariance matrix to be uncorrelated ^35^. Significant correlations between the scores of the means on any two principal components is an indication of correlated directional selection ^12^.

### Random matrices

**RandomMatrix():** Generating realistic random covariance matrices for null hypothesis testing is a challenging task, because random matrices must adequately sample the space of biologically plausible evolutionary parameters, like integration and flexibility. Most common covariance and correlation matrix sampling schemes fail at this, producing matrices with unrealistically low levels of integration, unless the level of integration is supplied *a priori* (as in 15). The function **RandomMatrix()** implements the method described in 46, which provides correlation matrices with a reasonable range of evolutionary characteristics. However, the adequacy of the generated matrices in hypothesis testing has not been well established, and we recommend these random matrices be used only for informal tests requiring an arbitrary covariance or correlation matrix.

## Summary

We have described a suite of functions dedicated to analyzing multivariate data sets within an evolutionary quantitative genetics framework. These functions focus on the central role that covariance and correlation matrices play in this framework; therefore, we provide functions that perform both descriptive statistics and hypothesis testing related to such matrices within an evolutionary context.

We have intentionally neglected to include techniques like phylogenetic regression or more extensive linear model functionality. Our reasons for this are twofold: the difficulty in transposing these methods efficiently to multiple traits, and the many different robust packages for performing some of these analyses, such as phytools, phylolm, pgls, nlme, MCMCglmm and others.

Some of the material implemented here is available in other sources or through custom implementations. We have attempted to create a single consistent source for these techniques. This is by no means an exhaustive effort, and we hope to expand it given demand from the community and further developments in the field. We hope to contribute to standardization and wide adoption of these tools, and, since we opted for an open source implementation under R, this also allows the involvement of the R community in using, debugging and complementing these tools, in an effort to contribute to an open scientific environment in which, for example, truly reproducible results are the norm rather than the exception.

## Software availability

The most recent version of the **EvolQG** package can be installed from GitHub using the package **devtools**:



                    > 
                    library
                    (devtools)
> 
                    install_
                    github ("lem-usp/evolqg")
                


A less up-to-date version is also available from CRAN:



                    > 
                    install.packages
                    ("evolqg")

1. Software available from: http://cran.r-project.org/web/packages/evolqg/
2. Latest source code: https://github.com/lem-usp/EvolQG
3. Archived source code as at time of publication: http://dx.doi.org/10.5281/zenodo.55121 ^63^
4. License: The MIT License ( https://opensource.org/licenses/MIT)
