# Supplementary material for: EvolQG - An R package for evolutionary quantitative genetics
Source: F1000Res. 2016 Nov 8;4:925. Originally published 2015 Sep 30. [Version 3] doi: 10.12688/f1000research.7082.3 (PMC5022708; doi:10.12688/f1000research.7082.3)
Supplement: Peer review history

# Peer review: Referee response for version 2

**DOI:** 10.5256/f1000research.9735.r16219

**Published:** 2016-09-12

**Authors:** Charles Roseman

**Affiliations:** 1 Department of Anthropology, University of Illinois at Urbana-Champaign, Urbana, IL, USA

Thank you for the opportunity to review the article “EvolQG - An R package for evolutionary quantitative genetics.” I am reviewing the version of 27 June 2016 (V.2). My overall impression of the paper and the associated package, which I have gone through in some detail, is that it makes a useful contribution to the study of the evolution of complex traits. Having many of these techniques available in an easily useable form will be a a boon to many researchers. I have some reservations that echo those of reviewers of previous and current versions of this manuscript.

 I think that a useful revision to the paper might be to clearly distinguish between applications and treatments of the P and G matrices. For morphological characteristics, I concur that there are good reasons to believe that P and G will be roughly proportional to one another (there are issues with what that might mean for evolutionary analyses), but handling errors in either case are rather different. The BootStrapRep() and MonteCarloRep() functions will work as described for a phenotypic correlation matrix, but not for a genetic correlation matrix, which would require some kind of resampling or simulation procedure that considered relatedness among individuals. This is not easy even in simple circumstances like those found in experimental studies (full-sib designs and the like) and very taxing in the kinds of pedigree based studies of wild organisms that are becoming the norm in the field.

  A work-around that I would accept only for analysis of legacy results from the literature would be to include a way to include effective sample sizes (Cheverud 1996, 1988). This would give an estimate of the number of effectively independently estimated breeding values worth of information in a sample and would be a better reflection of the errors in the genetic quantities. In the absence of this, adding explicit ways of working with the posterior distributions or estimated matrix of errors from the estimate of the G matrix would be welcome in your package. At the very least, you should make the distinction between these cases clearer in the text.

 I think the random skewers procedure should be revisited in one respect. The Marroig and Cheverud (2007) procedure describes drawing random selection gradient values from a uniform distribution and then standardizing the resulting vector to unit length. This is meant to provide random directions in the phenotypic space. This procedure, however, does not achieve this as the distribution of random points before standardization is from a hypercube of the same dimensionality as the number of traits. This will lead to an uneven distribution of points around the hypersphere on which the unit length vectors of standardized simulated selection gradients would occupy. In effect, the extra space in the corners of the hypersphere cause patches of excess vectors. This could cause considerable mischief if, for example, the first principal component of a covariance matrix was oriented directly at  corner of the hypercube. It might be worth modifying the procedure to draw random Gaussian variables, which are then standardized to unit length following Muller (1959) and Marsaglia (1972). This would ensure truly random simulated directional selection.

 I would like to second Prof. Houle’s point that there are many aspects of the different techniques that do not have much to do with evolution in an explicit way. Many of the matrix comparisons don’t have much to do with the degree to which responses to evolution might differ given a difference in the matrices. We could easily extend this critique to the parts of the package linking covariation to development. Phenotypic covariance/correlation matrices underdetermine the developmental processes through which covariation is generated and without some kind of independent assessment of developmental process. LModularity() arbitrarily assumes a lot about how organisms work. Without some extra information about the perturbations to developmental process, we have no idea what the biological meaning resulting clusters of traits might be or whether they relate to development at all.

 That said, I do think most of the techniques contained in the package will be very useful for many people. On balance, I regard EvolQG as a welcome contribution to the toolkit available to scholars interested in the evolution of complex traits. I would like to see the issues dealing with the errors in G and P to be clarified and the correction to the sampling in the random skewers to be implemented.

 Some smaller comments:

 “The Krzanowski shared space, or Krzanowski correlation, measures the degree to which the first principal components (eigenvectors) span the same subspace”

 This sentence and the section in which it is nested is a little opaque. Please reword so that it is clear how many principal components we need to consider.

 “transposing these methods efficiently to multiple traits, and the many different robust packages for performing some of these analyses..”

 “Transposing” does not strike me as the right word here. Please reword.

I have read this submission. I believe that I have an appropriate level of expertise to confirm that it is of an acceptable scientific standard, however I have significant reservations, as outlined above.

## References

[1] CheverudJ: A Comparison of Genetic and Phenotypic Correlations. *Evolution*.1988;42(5) : 10.2307/2408911 10.2307/2408911 28581166

[2] CheverudJ: Quantitative genetic analysis of cranial morphology in the cotton-top (Saguinus oedipus) and saddle-back (S. fuscicollis) tamarins. *Journal of Evolutionary Biology*.1996;9(1) : 10.1046/j.1420-9101.1996.9010005.x 5-42 10.1046/j.1420-9101.1996.9010005.x

[3] MarsagliaG: Choosing a Point from the Surface of a Sphere. *The Annals of Mathematical Statistics*.1972;43(2) : 10.1214/aoms/1177692644 645-646 10.1214/aoms/1177692644

[4] MullerM: A note on a method for generating points uniformly on n-dimensional spheres. *Communications of the ACM*.1959;2(4) : 10.1145/377939.377946 19-20 10.1145/377939.377946

# Response

**Published:** 2016-10-31

**Authors:** Diogo Melo

**Affiliations:** Universidade de São Paulo, Brazil

Thank you for the careful review! We respond to some of the specific comments below:

**I think that a useful revision to the paper might be to clearly distinguish between applications and treatments of the P and G matrices. For morphological characteristics, I concur that there are good reasons to believe that P and G will be roughly proportional to one another (there are issues with what that might mean for evolutionary analyses), but handling errors in either case are rather different. The BootStrapRep() and MonteCarloRep() functions will work as described for a phenotypic correlation matrix, but not for a genetic correlation matrix, which would require some kind of resampling or simulation procedure that considered relatedness among individuals. This is not easy even in simple circumstances like those found in experimental studies (full-sib designs and the like) and very taxing in the kinds of pedigree based studies of wild organisms that are becoming the norm in the field.**

 This is correct, and we have modified the text to make this clear.

**A work-around that I would accept only for analysis of legacy results from the literature would be to include a way to include effective sample sizes (Cheverud 1996, 1988). This would give an estimate of the number of effectively independently estimated breeding values worth of information in a sample and would be a better reflection of the errors in the genetic quantities.**

 I believe this is already possible in the package, if I understood correctly. Both BootstrapRep and MonteCarloRep have sample size arguments. 

**In the absence of this, adding explicit ways of working with the posterior distributions or estimated matrix of errors from the estimate of the G matrix would be welcome in your package. At the very least, you should make the distinction between these cases clearer in the text.**

 As much as we would like to provide general methods for G matrix error estimation, the current available mixed model packages in R for ML estimation make this difficult. We recommend using MCMCglmm or some other Bayesian framework to incorporate uncertainty, and the posterior samples can be trivially used with the functions in the package.

**I think the random skewers procedure should be revisited in one respect. The Marroig and Cheverud (2007) procedure describes drawing random selection gradient values from a uniform distribution and then standardizing the resulting vector to unit length. This is meant to provide random directions in the phenotypic space. This procedure, however, does not achieve this as the distribution of random points before standardization is from a hypercube of the same dimensionality as the number of traits. This will lead to an uneven distribution of points around the hypersphere on which the unit length vectors of standardized simulated selection gradients would occupy. In effect, the extra space in the corners of the hypersphere cause patches of excess vectors. This could cause considerable mischief if, for example, the first principal component of a covariance matrix was oriented directly at  corner of the hypercube. It might be worth modifying the procedure to draw random Gaussian variables, which are then standardized to unit length following Muller (1959) and Marsaglia (1972). This would ensure truly random simulated directional selection.**

 We generate random vectors using normal distributions for the elements to avoid this problem. Since the multivariate normal is spherically symmetrical, there are no privileged directions.

# Peer review: Referee response for version 2

**DOI:** 10.5256/f1000research.9735.r14607

**Published:** 2016-07-20

**Authors:** David Houle

**Affiliations:** 1 Department of Biological Science, Florida State University, Tallahassee, FL, USA

This article has been improved in response to the previous comments of the reviewers.  The authors have added several important and difficult-to-implement new analyses to the package, especially those discussed in Aguirre et al. 2014. The text  has been altered to make clearer the limitations of some of the procedures.  

 On the other hand, many of my more general comments about the shortcomings of the package still hold, and I refer the reader to my first set of comments. 

 One problem has, however, gotten worse, and that is how the package discusses the estimation of errors in G matrices and other variance component matrices. The text and code does not properly distinguish between sampling error of estimates of G matrices and sampling error of  simple statistics based directly on the data.   Consequently at many points the package could be used to produce misleading estimates of error in evolutionary statistics. 

 The section on matrix error and repeatability is the locus of this problem.  The opening paragraph in this section is about error in the G matrices, but none of the methods in that section actually apply to G matrices.  In particular  the text equates the REML-MVN method (Houle and Meyer, 2015) with this package's MonteCarloRep, but this is incorrect.  REML-MVN requires you to have an estimate of the sampling variance-covariance of matrix elements, but the software implements the assumption that the only parameter that is needed to construct an estimate of error is the sample size.  This works for a P matrix of multivariate normal data, but is NOT the REML-MVN method. 

 Similarly, the bootstrap routine seems only to bootstrap at the level of individuals.  Bootstrapping over individuals will always severely underestimate the error in G. For a designed experiment like a full and half-sib estimation of G, one could get proper estimates of error in G by bootstrapping over sires, which are the exchangeable units in that design.  A pedigree cannot be effectively bootstrapped as individuals are not exchangeable.  For a G matrix, you need additional estimates of error from either the posterior distribution of matrices, the variance-covariance matrix of estimates from a ML procedure, or  samples drawn from such a distribution.   Consequently the statement at the start of the Evolutionary Statistics section that **"MonteCarloStat()** and **BootstrapStat()** can be used to generate confidence intervals for these statistics" is incorrect. To properly deal with this problem all of the routines should allow the user to furnish samples of matrices from the sampling distributions (e.g. samples from the posterior distribution), and then produce measures of uncertainty from those.  

 It would be ideal for the package to implement the ability to utilize such samples in every procedure.

I have read this submission. I believe that I have an appropriate level of expertise to confirm that it is of an acceptable scientific standard, however I have significant reservations, as outlined above.

## References

[1] HouleDMeyerK: Estimating sampling error of evolutionary statistics based on genetic covariance matrices using maximum likelihood. *J Evol Biol*.2015;28(8) : 10.1111/jeb.12674 1542-9 10.1111/jeb.12674 26079756

[2] AguirreJDHineEMcGuiganKBlowsMW: Comparing G: multivariate analysis of genetic variation in multiple populations. *Heredity (Edinb)*.2014;112(1) : 10.1038/hdy.2013.12 21-9 10.1038/hdy.2013.12 23486079PMC3860158

# Response

**Published:** 2016-10-31

**Authors:** Diogo Melo

**Affiliations:** Universidade de São Paulo, Brazil

Thank you for the second careful review! We agree completely with the problems regarding G-matrix error and have modified the text to make these limitations clear. As much as we would like to provide general methods for G matrix error estimation, the current available mixed model packages in R for ML estimation make this difficult. We recommend using MCMCglmm or some other Bayesian framework to incorporate uncertainty.

# Peer review: Referee response for version 1

**DOI:** 10.5256/f1000research.7623.r10610

**Published:** 2015-11-16

**Authors:** David Houle

**Affiliations:** 1 Department of Biological Science, Florida State University, Tallahassee, FL, USA

This paper describes the functions implemented in the R package “evolvqg.”  This package implements a large set of methods for characterizing and comparing variance-covariance matrices. A compendium of such methods is useful, but the user should be aware of shortcomings in this package. 

First, the authors justify grouping these methods together under the umbrella of “evolutionary quantitative genetics.” Under this label, the very diversity of methods is a bit misleading - the majority of implemented methods have no explicit relationship to evolution, and this is not clear in the paper. To a large degree this confusion reflects the state of the field. A great many ad hoc methods are proposed and widely applied, such as the popular Mantel test. For example, this package implements methods for quantifying repeatability of the phenotypic covariance and correlation matrices, which is a fine thing to do, but has little relevance to the genetic matrices that underlie evolution. For those matrices, any procedure for estimating them will partition out the non-genetic factors as part of the hierarchical model fitting. Repeatability is usually the least of our estimation problems. Other methods that are in this not-really-evolutionary category include, matrix correlations, the ‘modularity’ analyses. 

A related issue is that this package does not include state-of-the-art techniques. In particular, the advent of MCMC methods (plus other methods for getting sampling variation of matrices: Houle and Meyer 2015) provides proper measures of uncertainty for the evolutionarily relevant G matrices. The previously available R package “evolvability” uses the posterior distributions for G matrices as well as selection gradients, when available, to place confidence intervals on the Hansen and Houle measures of evolvabilities. The “evolqg” package under review here primarily implements methods developed and used by the Marroig group, lacking some of the most promising approaches and metrics developed by others (e.g. Hine *et al*. 2009, Houle and Fierst 2013; Aguirre *et al*. 2014). Programming time is of course a limitation, and no one is under obligation to implement everything. However, it is deficient scholarship that this paper makes no mention of these approaches, or of the availability of software that does these analyses.  

Based on their descriptions here, the phylogenetic comparison methods implemented in this package seem to be quite deficient. AncestralStates is contradictorily described as dealing with multivariate data, but reconstructs each character independently. It appears to implement an interface to a univariate method, and is not actually a multivariate approach. PhyloW and PhloCompare compute “weighted” estimates of matrices at internal nodes, but the proper weight to apply to a G matrix is not clear at all. This appears to only deal with sampling variance at the individual level, neglecting the more important sources of matrix variation. This disconnect appears to rest on the author's assumption that “As a general rule, high similarity between populations’ P-matrices is a good indicator of high similarity between P and G”. This is certainly not a general rule, as P and G matrices for traits with low heritability can be very different from each other. The classic examples are life history traits.

For drift models, this implementation shares a major deficiency with previous work, in assuming that the expectation is that matrices will remain proportional. This is indeed the large-sample expectation, but any actual population will deviate from that expectation in ways that depend on the unknown parameters of the underlying system – the number of loci, their relative mutability, the underlying M matrix, and of course the effective population size of each part of the genome. For example, Griswold *et al*. 2007 show that even when the underlying M matrix is spherical, realizations based on this will have substantial deviations from sphericity. There is no general treatment of this problem, so all results from such analyses need to be treated with caution. A failure to reject a departure from proportionality is meaningful, but rejections of proportionality do not necessarily indicate that drift is not responsible.

Finally, like much of the software being made freely available, there is no description of what the authors did to validate their implementation of these techniques, and no comparisons with previous analyses to indirectly validate them. One or both of these should really be standard with new software. I know that it is not standard, and do not want to single these authors out on that account. The user, however, should be aware of all unvalidated software, and should perform their own checks. Unfortunately, this is only easy for simple procedures for which a package is not really necessary.

Some very specific issues:

1. Errors: “The proportion of variance not associated with the individuals is called the repeatability.”
2. The description of Mantel tests is misleading, as a high correlation does not mean that matrices are the “same,” nor does a negative correlation mean that matrices are “opposite.”
3. The description of the PCA similarity algorithm is opaque. What is “pondering”? 

I have read this submission. I believe that I have an appropriate level of expertise to confirm that it is of an acceptable scientific standard, however I have significant reservations, as outlined above.

## References

[1] GriswoldCKLogsdonBGomulkiewiczR: Neutral evolution of multiple quantitative characters: a genealogical approach. *Genetics*.2007;176(1) : 10.1534/genetics.106.069658 455-66 10.1534/genetics.106.069658 17339224PMC1893077

[2] HineEChenowethSFRundleHDBlowsMW: Characterizing the evolution of genetic variance using genetic covariance tensors. *Philos Trans R Soc Lond B Biol Sci*.2009;364(1523) : 10.1098/rstb.2008.0313 1567-78 10.1098/rstb.2008.0313 19414471PMC2691006

[3] HouleDMeyerK: Estimating sampling error of evolutionary statistics based on genetic covariance matrices using maximum likelihood. *J Evol Biol*.2015;28(8) : 10.1111/jeb.12674 1542-9 10.1111/jeb.12674 26079756

[4] HouleDFierstJ: Properties of spontaneous mutational variance and covariance for wing size and shape in Drosophila melanogaster. *Evolution*.2013;67(4) : 10.1111/j.1558-5646.2012.01838.x 1116-30 10.1111/j.1558-5646.2012.01838.x 23550760

[5] AguirreJDHineEMcGuiganKBlowsMW: Comparing G: multivariate analysis of genetic variation in multiple populations. *Heredity (Edinb)*.2014;112(1) : 10.1038/hdy.2013.12 21-9 10.1038/hdy.2013.12 23486079PMC3860158

# Response

**Published:** 2016-06-13

**Authors:** Diogo Melo

**Affiliations:** Universidade de São Paulo, Brazil

***- This paper describes the functions implemented in the R package “evolqg.”  This package implements a large set of methods for characterizing and comparing variance-covariance matrices. A compendium of such methods is useful, but the user should be aware of shortcomings in this package.***

 Thank you for your insightful comments. One of the reasons we chose F1000research for this article is our perception that something is amiss in the current system of peer-review, where good reviewers are burdened and swamped by manuscripts and authors tend to often ignore their criticisms and take the short road to the next journal inline instead of dealing with the criticisms and polish their manuscripts. The fully open system implemented by F1000 seems like a good way out of this trap we express our gratitude to Drs. Houle and Grabowsky for taking their time to criticize this package.

 We respond to comments individually after each of your considerations.

***- First, the authors justify grouping these methods together under the umbrella of “evolutionary quantitative genetics.” Under this label, the very diversity of methods is a bit misleading - the majority of implemented methods have no explicit relationship to evolution, and this is not clear in the paper. To a large degree this confusion reflects the state of the field. A great many ad hoc methods are proposed and widely applied, such as the popular Mantel test. For example, this package implements methods for quantifying repeatability of the phenotypic covariance and correlation matrices, which is a fine thing to do, but has little relevance to the genetic matrices that underlie evolution. For those matrices, any procedure for estimating them will partition out the non-genetic factors as part of the hierarchical model fitting. Repeatability is usually the least of our estimation problems. Other methods that are in this not-really-evolutionary category include, matrix correlations, the ‘modularity’ analyses.***

 This is a fair comment, and indeed an argument could be made to separate the different aspects of evolutionary research that are presented here into different packages. We chose to keep these different methods in a single source since we feel a consistent workflow is very beneficial to research on evolution and covariation. For example, many of the latter hypothesis-testing methods presented in our paper should only be used if some level of matrix similarity is detected, and the matrix similarities, in turn, should not be interpreted without matrices' repeatabilities. Furthermore, while the development of multivariate evolutionary theory and the theory of integration and modularity were separate, Lande's, Cheverud's and Wagner's work since the 80s have linked these fields very intimately, to a point that it is hard for us to think of these fields as separate. From our point of view, the influence of covariation in evolution, and the genetic and developmental origin of these variational associations make integration and modularity central to modern evolutionary theory, even though admittedly there are a number of researchers who consider morphological integration and comparative quantitative genetics as two separate fields. We feel this integrative approach links the developmental and intra-populational causes of genetic covariation to their evolutionary consequences, leading to a more complete and robust understanding of micro- and macro-evolution.

 Finally, the inclusion of methods that are mostly "statistical" and not "evolutionary" should make it easier for researchers to check the quality of their data and its appropriateness for further analyses.

***- A related issue is that this package does not include state-of-the-art techniques. In particular, the advent of MCMC methods (plus other methods for getting sampling variation of matrices: Houle and Meyer 2015) provides proper measures of uncertainty for the evolutionarily relevant G matrices. The previously available R package “evolvability” uses the posterior distributions for G matrices as well as selection gradients, when available, to place confidence intervals on the Hansen and Houle measures of evolvabilities. The “evolqg” package under review here primarily implements methods developed and used by the Marroig group, lacking some of the most promising approaches and metrics developed by others (e.g. Hine et al. 2009, Houle and Fierst 2013; Aguirre et al. 2014). Programming time is of course a limitation, and no one is under obligation to implement everything. However, it is deficient scholarship that this paper makes no mention of these approaches, or of the availability of software that does these analyses.***

 While we challenge the reviewer's assertion that we fail to mention these methods (see the second paragraph of our Summary section; and the method described in Houle and Meyer is implemented by the function MonteCarloStat()), the point is well taken. Another reason for choosing F1000Research was the ease of updating the manuscript as we add new functionality to the EvolQG package. In this spirit of continuous development, we added in the revised version of the package three of the methods described in Aguirre et al. 2014, including the eigentensor decomposition described by Hine et al. 2009, using fast and flexible implementations in R.

***- Based on their descriptions here, the phylogenetic comparison methods implemented in this package seem to be quite deficient.***

 We would argue that the available methods in the literature are deficient in dealing with multivariate correlated traits, and we provide the rather simple available methods.

***- AncestralStates is contradictorily described as dealing with multivariate data, but reconstructs each character independently. It appears to implement an interface to a univariate method, and is not actually a multivariate approach.***

 We clearly stated that AncestralStates is not a multivariate approach. Indeed AncestralStates is just a wrapper to facilitate reconstructing multiple traits independently. If the reviewer has a suggestion on how to implement this taking the multivariate covariance structure into account for many traits we would be very interested, but this remains an active research topic, and we have not found a satisfactory solution for this problem. Since this seems to be more misleading than helpful, we have removed this function from the package and manuscript.

***- PhyloW and PhyloCompare compute “weighted” estimates of matrices at internal nodes, but the proper weight to apply to a G matrix is not clear at all. This appears to only deal with sampling variance at the individual level, neglecting the more important sources of matrix variation.***

 This is another unambitious method, and was intended only to calculate within-group phenotypic covariance matrices in a phylogenetically structured way. The use with G-matrices coming from more complex linear models would indeed be non-trivial, and we now make this explicit in the description.

***- This disconnect appears to rest on the author's assumption that “As a general rule, high similarity between populations’ P-matrices is a good indicator of high similarity between P and G”. This is certainly not a general rule, as P and G matrices for traits with low heritability can be very different from each other. The classic examples are life history traits.***

 We would argue that in the case of low heritability the P-matrices between populations would also be dissimilar. The point here is not that P and G are always similar, but that similar Ps between populations are a fair indication of similar Gs and Ps, at least in the groups we have worked with. In mammals, this conclusion is supported empirically by the comparisons of 5 different G-matrices with P-matrices of several groups, which indicate similar responses to random selection on average (Porto, 2009). In any event, we add a caveat on the function description that structurally similar matrices are a key component of the methods implemented here.

***- For drift models, this implementation shares a major deficiency with previous work, in assuming that the expectation is that matrices will remain proportional. This is indeed the large-sample expectation, but any actual population will deviate from that expectation in ways that depend on the unknown parameters of the underlying system – the number of loci, their relative mutability, the underlying M matrix, and of course the effective population size of each part of the genome. For example, Griswold et al. 2007 show that even when the underlying M matrix is spherical, realizations based on this will have substantial deviations from sphericity. There is no general treatment of this problem, so all results from such analyses need to be treated with caution. A failure to reject a departure from proportionality is meaningful, but rejections of proportionality do not necessarily indicate that drift is not responsible.***

 This is indeed a problem, and we try to remedy this by ensuring the matrices share some minimum level of similarity before using these drift models. Proa et al. 2013 analysed the type I error rate in the DriftTest method, and found that if matrices are similar the test is well behaved. This similarity must be tested on a case by case basis. Error rate analysis of the other tests is an open problem. Another option is to repeat the analysis using different matrices from the terminal taxa that represent extremes of variability, and check if the results are robust to this. We make these caveats and problems clear in the revised manuscript.

 This paragraph now reads:

 "Since both these tests use drift as a null hypothesis, failure to reject the null hypothesis is not evidence that selection was not involved in the observed pattern of diversification, only that the observed pattern is compatible with drift. Also, these methods assume that the matrices involved share some degree of similarity, and should ideally be proportional to each other. We would be very weary of using these methods if the matrices are too dissimilar, or if the results change radically if different matrices are used as the ancestral matrix. Also, these tests rely on two levels of replication, taxa and traits. As a general guideline, at least 20 traits and at least 8 taxa should be sampled for using these methods with any confidence, and results should be analyzed in conjunction with other lines of evidence."

 With regards to Griswold et al. 2007, we believe that verifying the extant matrices are similar somewhat sidesteps these problems, and in any event their simulations do not include stabilizing selection on covariance patterns, which is very likely to exist if matrices are stable in evolutionary timescales. It’s important to realize that the methods we describe are for identifying drift on species means, not covariances. Evolution of covariance patterns is a different matter altogether.

***- Finally, like much of the software being made freely available, there is no description of what the authors did to validate their implementation of these techniques, and no comparisons with previous analyses to indirectly validate them. One or both of these should really be standard with new software. I know that it is not standard, and do not want to single these authors out on that account. The user, however, should be aware of all unvalidated software, and should perform their own checks. Unfortunately, this is only easy for simple procedures for which a package is not really necessary.***

 We agree entirely, and feel that the bar for scientific software should be high, and so we took additional steps in this direction. While no implementation is bug free, we compared all results from our initial set of functions between different implementations done by members of our lab and available implementations in the literature. Also, all development for the package was done in a test driven development framework, and all functions have unit tests for the most or all of their functionality, that is run every time the package is built. This insures modifications do not alter previous results. The implementations in the package follow a modular design for most functionality, minimizing code duplication and reducing the chance of bugs. We also have a fast and constantly maintained issue and bug tracker in github, where users can ask questions, request new functionality, and report bugs.

***- Some very specific issues:***

***- Errors: “The proportion of variance not associated with the individuals is called the repeatability.”***

 Changed to something clearer. Now reads: "The proportion of variance associated with among individual variation, and not within individual variation, is called the repeatability"

***- The description of Mantel tests is misleading, as a high correlation does not mean that matrices are the “same,” nor does a negative correlation mean that matrices are “opposite.”***

 We chose a more cautious wording of these general guidelines. Now reads: "The correlation between matrices range between -1 and 1, and higher correlations indicate matrices have more similar structures, while null correlations indicate the matrices have very distinct correlation structures. Correlations near zero can also occur if the elements of the matrices have nonlinear relationships between them, as in all Pearson correlations. Negative correlations indicate the pattern of association between traits is reversed in the two matrices."

***- The description of the PCA similarity algorithm is opaque. What is “pondering”?***

 Sorry, this was a rather hard false cognate with portuguese for us to catch, and now reads:  "In order to take the variation into account, we can add the eigenvalue associated with each principal component into the calculation, effectively weighting each correlation by the variance in the associated directions"

 Literature cited

1. Cheverud, J. M. (1996). Quantitative genetic analysis of cranial morphology in the cotton‐top (Saguinus oedipus) and saddle‐back (S. fuscicollis) tamarins. Journal of Evolutionary Biology, 9(1), 5-42.
2. Marroig, G., & Cheverud, J. M. (2001). A comparison of phenotypic variation and covariation patterns and the role of phylogeny, ecology, and ontogeny during cranial evolution of New World monkeys. Evolution, 55(12), 2576-2600.
3. Prôa, M., O'Higgins, P., & Monteiro, L. R. (2013). Type I error rates for testing genetic drift with phenotypic covariance matrices: a simulation study. Evolution, 67(1), 185-195.
4. Porto, A., de Oliveira, F. B., Shirai, L. T., De Conto, V., Marroig, G., (2009). The Evolution of Modularity in the Mammalian Skull I: Morphological Integration Patterns and Magnitudes. Evolutionary Biology, 36(1), 118–135. doi:10.1007/s11692-008-9038-3

# Peer review: Referee response for version 1

**DOI:** 10.5256/f1000research.7623.r10607

**Published:** 2015-10-16

**Authors:** Mark Grabowsky

**Affiliations:** 1 Centre for Ecological and Evolutionary Synthesis (CEES), Department of Biosciences, The Faculty of Mathematics and Natural Sciences, University of Olso, Oslo, Norway

This Software Tool is an open source R package for performing a wide variety of evolutionary genetic analyses. It comes at a particularly useful time when large data sets required to use these analytical techniques are more and more available. I predict this package will be used to a greater and greater extent over time.

The team, with lead author Diogo Melo, has made a number of major innovations in the field of evolutionary quantitative genetics and this software tool is well within their purview.

The group describes the potential application of the new software well, cites the sources for the development of the different methods included, and briefly explains each function with an adequate level of discussion. I found the section on Phylogenetic Comparisons most interesting, and would have liked more discussion of these points.

Issues:

1. It would be nice to include data for which the tools could be tried out on and examples of the output included in the text.
2. I also worry about the power of some of these tests, and would appreciate if the authors were to think about adding in caveats where available. There are a few papers out currently that address these issues (e.g. Haber, 2011). For example, I have both heard and performed some analyses using another version of the DriftTest() function and it appears about 20 traits are required to ever reject the null hypothesis. As this package is sure to be used by researchers who are less acquainted with these issues they may be unaware of issues with sample sizes, etc.

I have read this submission. I believe that I have an appropriate level of expertise to confirm that it is of an acceptable scientific standard.

# Response

**Published:** 2016-06-13

**Authors:** Diogo Melo

**Affiliations:** Universidade de São Paulo, Brazil

Thank you for your kind comments. We have included some additional caveats in relation to the power and level of replication required for some of the tests. With regards to examples, we fell a package vignette tutorial is much more suited to this, and we are working on more documentation to be distributed with the package.
